# Osmium isotope analysis as an innovative tool for provenancing ancient iron: A systematic approach

**DOI:** 10.1371/journal.pone.0229623

**Published:** 2020-03-18

**Authors:** Michael Brauns, Naama Yahalom-Mack, Ivan Stepanov, Lee Sauder, Jake Keen, Adi Eliyahu-Behar

**Affiliations:** 1 Curt-Engelhorn-Zentrum Archäometrie, Mannheim, Germany; 2 Institute of Archaeology, The Hebrew University, Jerusalem, Israel; 3 Institute of Archaeology, Ariel University, Ariel, Israel; 4 Independent Researcher, Lexington, Virginia, United States of America; 5 Independent Researcher, Wimborne, Dorset, United Kingdom; 6 The Department of Chemical Sciences, Ariel University, Ariel, Israel; University at Buffalo - The State University of New York, UNITED STATES

## Abstract

The innovation of iron production is often considered one of the greatest technological advances in human history. A reliable provenancing method for iron is instrumental for the reconstruction of economic, social and geo-political aspects of iron production and use in antiquity. Although the potential of osmium isotopes analysis for this purpose has been previously suggested, here we present for the first time the results of osmium isotope analysis of ores, bloom and metal obtained from a set of systematic, bloomery iron-smelting experiments, utilizing selected ores from the Southern Levant. The results show that the ^187^Os/^188^Os ratio is preserved from ore to metal, with no isotopic fractionation. In addition, enrichment/depletion of osmium content was observed in the transition from ore to metal and from ore to slag. This observation has potential significance for our ability to differentiate between the various processes and sheds light on the suitability of various production remains for this method, which emerges as a robust and promising tool for the provenancing of archaeological ferrous metals.

## Introduction

Sourcing raw materials has the potential to shed light on social, political and economic aspects of past societies. Metals in particular, which are not equally distributed geographically, were often obtained from afar. This required a developed system of trade, organization and long-distance contacts. These labor-intensive investments, along with the intrinsic high value and prestige nature accorded to metals, led to the concentration of this valuable commodity in the hands of elites. The ability to compare a metal product to its ore source is therefore highly instrumental in reconstructing significant aspects of ancient society and economy. The provenancing of metals heavily relies on the application of geochemical and analytical techniques, accompanied by the compilation of a database of geological ore sources. While chemical and lead isotope analysis (LIA) are routinely applied for the provenancing of lead, silver and copper-based alloys [1,2], the provenancing of iron has remained limited, due to the current available methodology.

### Iron provenancing methodology and osmium analysis

Iron provenancing studies have focused mainly on the chemical analysis of metal and particularly the slag inclusions which remain in the metal following the smelting process. Emphasis is placed on the correlation of major and trace element concentrations in the slag inclusions with that of the ore and/or the smelting slag [3–12]. At present, the most-developed methods rely on the combination of X-ray microanalysis and laser ablation coupled mass spectrometry [9,12]. One major disadvantage of these analyses is the destructive nature of the sampling, requiring sectioning and exposing a large surface area of the item under study. Moreover, the analytical procedure is limited by the size of the inclusions, depending on the diameter of the laser beam (a minimum spot size of 30–50 mm is commonly used to obtain a sufficient count rate [9]).

Leroy and co-authors [12], for example, studied the circulation of iron ores and products in medieval Ariège in the French Pyrenees. Based on the above-mentioned analytical procedures and in combination with multivariate statistical methods, they clarified some of the procurement patterns already known from historical documents. However, apart from a few geographically localized case studies such as this one, the success of these methods is limited, particularly when the starting point–the ore source–is unknown and where historical sources do not exist.

Several attempts were made to compare the isotopic signature of iron objects and slag to that of ores. Lead (Pb), strontium (Sr), iron (Fe) and osmium (Os) isotopes have all been employed to investigate their suitability for iron provenancing, with varying levels of success [13–17]. Brauns et al. [15] argued that lead isotopic ratios are probably of limited value for sourcing iron, as their inhomogeneity in many iron ore deposits is even higher than for most non-ferrous ores due to generally low lead and high uranium concentrations [13]. Moreover, as lead is an element with siderophile-chalcophile affinities, it partitions between slag and metal so that the lead concentrations in iron artefacts are often too low to determine the isotopic ratios. Adding the use of strontium in combination with lead isotopes was based on similar methodologies applied for glass provenance [18]. Strontium is a lithophile element and thus will be preferentially taken up by the silicate slag. However, strontium is also one of the most abundant elements in nature, so that the strontium signature of an ore may be easily influenced by a small admixture of strontium bearing components such as charcoal, lime or clay involved in the iron production process [15].

The use of iron isotopes for provenancing ferrous archaeological metals has been employed as well [16,17]. However, determining the provenance of heavily corroded iron objects, which is often the case of early Iron Age artifacts from the Levant [19], is near impossible due to isotope fractionation during corrosion [20–22]. In order to address this obstacle, Brauns and the co-authors [15], based on a limited dataset (13 analyses), suggested that the osmium isotopic composition (Os IC) remains unchanged throughout the various stages of the iron production process–a feature which renders Os IC a good candidate for iron provenancing. This suggestion, if securely verified on a statistically significant dataset, makes provenancing of ancient archaeological iron based on Os IC a s significant contribution.

Osmium has seven naturally occurring isotopes: ^184^Os, ^186^Os, ^187^Os, ^188^Os, ^189^Os, ^190^Os, ^192^Os. Of these, ^187^Os is the daughter isotope of ^187^Re (half-life 4.56×10^10^ years) and is most often expressed as ^187^Os/^188^Os. Osmium has proven to be an excellent tracer in environmental research projects and in earth sciences, in tracing, for example, crustal contamination of mantle-derived melts [23–25]. The highly siderophile geochemical nature of this element, which results in an enrichment of osmium in the metal phase, renders osmium a formidable tracer in archeometallurgical studies of iron.

### Early iron production

Provenancing iron is exceptionally important when it becomes prevalent in daily use. In the southern Levant this occurred in the early first millennium BCE, with the rise of Iron Age territorial kingdoms, such as Judah and Israel [26,27]. The control of iron sources likely was a strategic military and economic advantage at that time.

In antiquity, in most parts of the world, iron was directly obtained by reducing the ore in a small pit or furnace via the bloomery process–the oldest iron-smelting method known, which was continuously in use until the late Medieval period. Archaeological evidence for the bloomery process has been identified at major Iron Age sites in the Southern Levant, for example, at Hazor, Megiddo, and Tell es-Safi-Gath in Israel and Tell Hammeh, in Jordan. In all these sites, iron production was dated to the early first millennium BCE (Iron Age IIA, late 10th—early 9th centuries), and was often associated with administrative buildings, suggesting that it had been controlled by a central authority[28–31,27,32,33]. This marks a departure from the predominant mode of bronze production that prevailed until the advent of iron, which was mostly characterized by local independent workshops.

**Fig 1:**
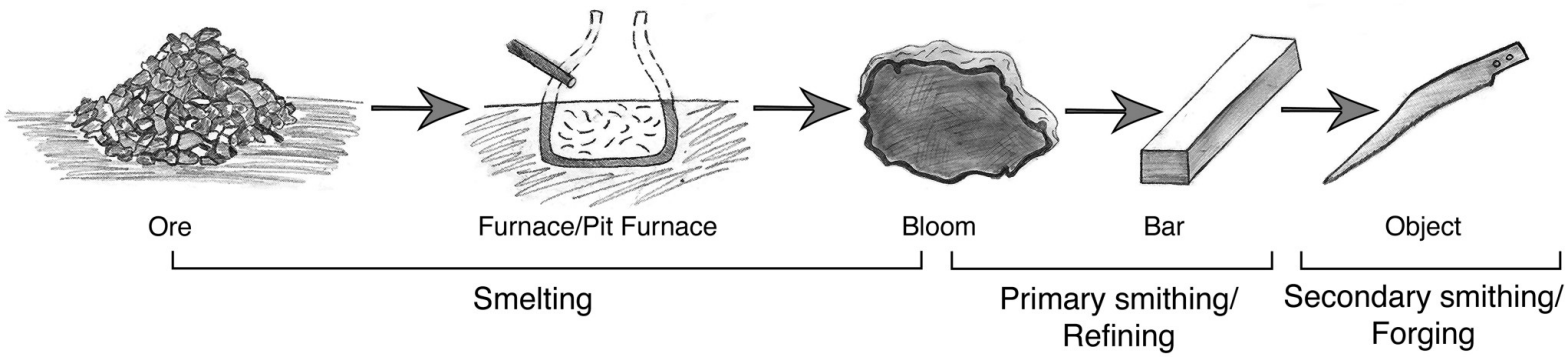
A schematic representation of the ‘chaîne opératoire’ of iron production. The figure shows the various stages of the process, including ore crushing and smelting, and the various products, including bloom, bar, and object, formed through primary and secondary smithing, (modified from Eliyahu-Behar et al. 2013).

Generally, the process can be roughly divided into three main stages: the smelting (reduction) of the ores to produce a bloom (a spongy mass that is a mixture of metal and slag), the refining of the bloom (primary smithing) to produce a more compacted metal (a bar ingot) and the forging of the end product (secondary smithing, see Fig 1). During smelting, the furnace was continuously charged with iron ore and charcoal, placed in alternating layers. A draft of air was directed through tuyères to the lower part of the furnace. The temperature in the hot-zone, the working area, was not sufficiently high (1100–1350°C) to melt the iron, thus reduction was obtained in the solid phase. The silicate phase, i.e. the slag did however liquefy at these temperatures and was either tapped out of the furnace or left to cool inside it (forming a furnace slag, also known as a slag cake). The resulting product—the bloom—was removed from the fire and hammered to consolidate the iron and to squeeze out some of the remaining slag. The by-product of this stage (primary and secondary smithing), is denoted ‘smithing slag’.

It has often been assumed that since iron is the second most-abundant metal in the earth’s crust and since it tends to outcrop at the surface, it was readily available. In comparison to other metals, particularly the copper which iron eventually replaced for mundane purposes, mainly agriculture and warfare, it was indeed more abundant, but not as much as once thought. With the existing technology (the bloomery process), it was necessary to use rich, high-grade iron ores, since during reduction, a great amount of iron is lost into the slag. This results in an extremely low yield; under usual reduction conditions (1100–1350°C), an ore with an iron-to-silica ratio lower than three may result in zero free iron, and therefore no yield at all [7].

### Iron ore deposits in the southern Levant

**Fig 2:**
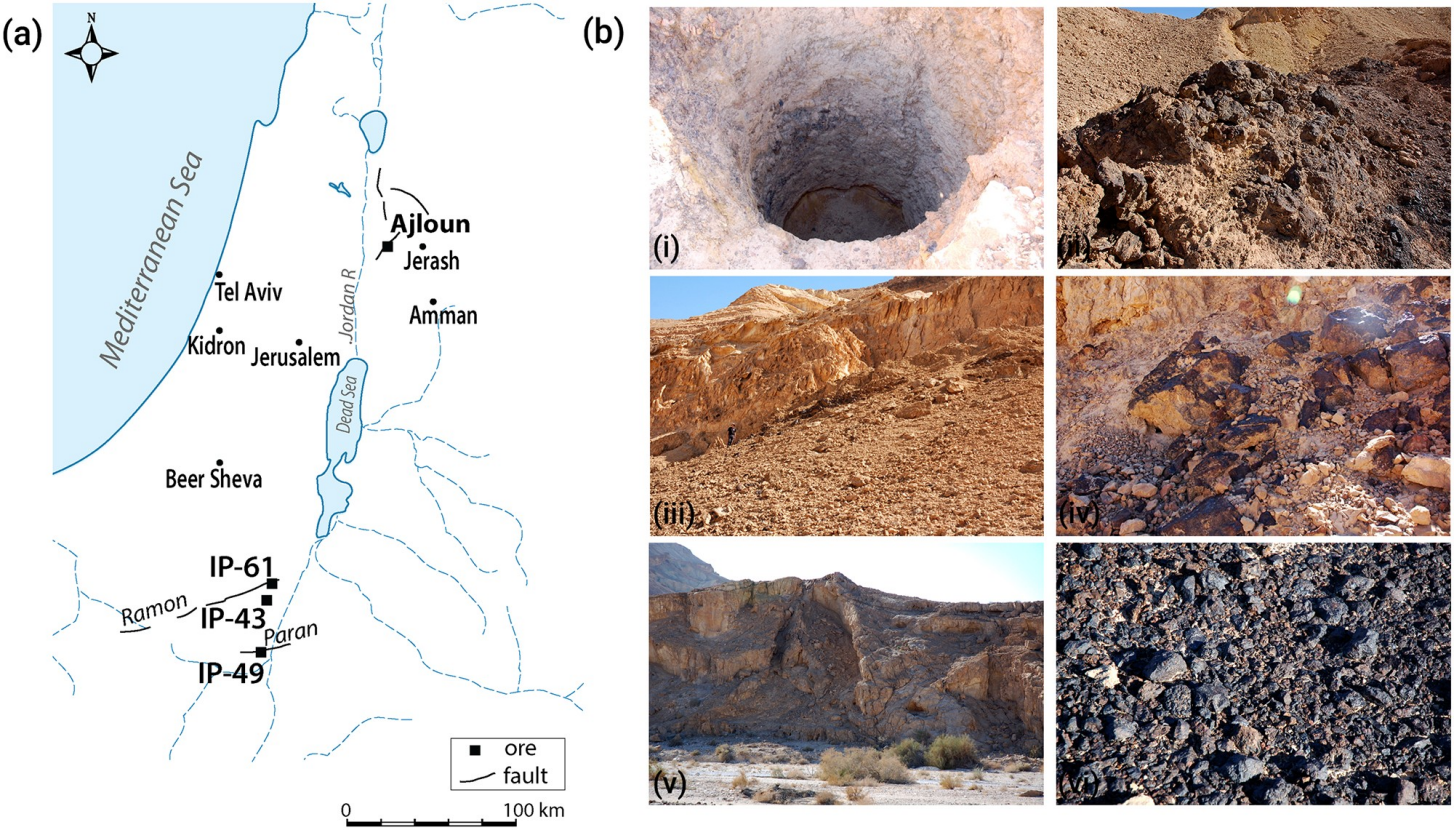
Iron ore sources analyzed in the study. (A) Map of southern Levant, showing the location of the iron ores used in the study. Also shown are geological faults lines in the vicintiy of the ore depostis (ref: GSI website). (B) Field views of the ores used for the smelting experiments; i-ii, Zavar IP49, iii-iv, Nekarot IP61, v-vi, Nekarot-Evus IP43.

In the southern Levant, and specifically in modern-day Israel, high-quality iron-rich ore deposits are limited. In the framework of ore prospecting initiated by the Israeli government during the 1950s, iron-rich epigenetic mineralization was identified along major trending faults related to the Dead Sea Transform (DST) in the Negev region in Israel and Jordan (Fig 2). Several mechanisms were suggested for the formation of these ores, and studies of their paragenesis show variability in the associated minerals and especially in their iron content. [34,35]. The iron content has a particular significance, given their potential for bloomery exploitation in antiquity; however, no archaeological evidence for their exploitation has been so far identified.

Apart from the iron mineralization known in the northern Negev in southern Israel, hypogene iron ores also occur in the Ajloun region in northwestern Jordan, where *Mugharet el-Wardeh* is the largest deposit (Fig 2). In contrast to the Negev deposits, evidence of exploitation of the *Mugharet el-Wardeh* ore is known from the Iron Age, possibly continuing throughout the Islamic period [28,36,37]. The iron content and mineralogy in the Negev and the Ajloun is similar, both containing an average of 70–85 wt% iron oxide, and mainly hematite and goethite (Fe_2_O_3_/FeOOH), as well as calcite and quartz as associated gangue minerals [34,36].

## Materials and methods

### Iron ores

Rich ore deposits were identified in the northern Negev region of southern Israel. Following laboratory smelting experiments and analysis (forthcoming), three ore sources were sampled for the bloomery field experiment (Field permit No. 138/2019 south from Israel Nature and Parks Authority. https://www.parks.org.il/en/). The three were sampled for two main reasons; showing good bloom consolidation degree in laboratory trials and being easily accessible for collection of high quantity of ore. Rich iron ores from the Ajloun region in Jordan, previously published [36,38], were sampled for their Os IC. The ore deposits sampled for this study are presented in Table 1 and Fig 2.

**Table 1:** Iron ores. The location, coordinates and mineralogy of iron ores used in this study.

Experiment No.	Ore No.	Ore Name	Location	Location(GPS coordinates)	Average Iron oxide (Fe_2_O_3)_ (*measured in this study)	Mineralogy (major)
EXP-1	IP-49	Zavar	Wadi Paran (prospected by GSI)	35.024059; 30.317927	80%	Hematite, Goethite
EXP-5	IP-43	Nekarot-Evus	Wadi Nekarot/on the incense route.	35.041301; 30.573553	88%	Hematite, Goethite
EXP-6	IP-IP61	Nekarot	Wadi Nekarot	35.078211; 30.644197	63%	Goethite
	IP-27-28/IP-64-66	Ajloun	Ajloun	32.222414; 35.713313	78%	Mostly Hematite

The sampled iron mineralizations can be generally described as ferruginous lenses and iron oxide vein stockworks formed along major faults in the Upper Cretaceous. Sample IP-49 (*Zvar Habaqbuq*) is located along the eastern part of the *Menuha* ridge segment of one of the major faults (*the Paran fault*) and are hosted by Middle to Late Cretaceous carbonate sequences of the Judea Group. The deposit appears between the *Tamar and Ora formations* and is comprised of three main mineralization; hematite, goethite and a hematitic-jasperoids. Associated secondary minerals are mainly calcite, dolomite and to a lesser extent gypsum and barite [34,35]. Sample IP-43 and Sample IP-61, were both collected from *Wadi Nekarot*, associated with the *Ramon fault* (Fig 2). Both are localized mineralization similar to that of IP-49 and are hosted by the same rocks [34].

The iron ore body *of Mugharet el-Wardeh*, in the Ajloun Region in Jordan has a lenticular or vein-like shape, measuring ca. 300 m in length and up to 10 m in width. Similarly, it was formed along faults and fractures resulting from tectonic activity associated with the Dead Sea Transform (DST). The main mineralization consists of hematite as a major component, along with minor percentages of limonite, goethite, calcite, quartz, and chalcedony. An average of 68 wt% of iron oxide (Fe_2_O_3_) was previously measured [36,38].

### Smelting experiments

The smelting experiment was conducted during February 2019, in Mesheq Hanan (Kidron, Israel), a farmstead equipped with the necessary facilities, as well as a modern iron workshop (Fig 2). Experiments were conducted and overseen by two professional smelters, Mr. Jake Keen (Britain) and Mr. Lee Sauder (USA). The experiments involved building a shaft furnace, preparing and roasting the ores, smelting the ores to produce a bloom and forging the bloom into a semi-product–an iron bar–thus performing the technical part of the *chaine opératoire* of ancient iron production. The shaft furnace was built from a mixture of kaolinite commercial clay mixed with building sand and straw (in app. 13:33:1 ratio by weight). The clay structure was built against a round column of bamboo, built row by row, tying string around each row of clay to keep it from slumping outwards. Prior to operation, the furnace was dried out with a fire set in its interior, burning the bamboo inner structure.

Ores were beneficiated and prepared for smelting by crudely crushing the chunks to assess their quality; iron-rich chunks were selected through visual examination. Roasting in a wood fire was subsequently performed, placing the ore and wood in alternating layers. The fire was maintained for several hours and the heap was left to cool. After roasting, ores were crushed into “pea-sized” pieces, 1–2 cm in size. Commercially available charcoal (imported from Colombia) that was used as fuel had been crushed to pieces ranging 5–10 cm. (All the materials involved in the process were characterized for their Os IC, see below).

The temperature was regulated by pushing air using an electric bellow attached to a copper tuyère. The tuyère was placed at an angle of 17 degrees at about 25 cm above the base of the furnace. Throughout the operation, the temperature was recorded in intervals, using thermocouples fixed in four positions along the height of the furnace, at 90 degrees relative to the tuyère.

The three smelts were conducted under similar conditions, using roughly the same parameters of air flow, temperature and loading intervals. After preheating the furnace with wood and charcoal, we began loading the furnace with alternating layers of roasted ore and charcoal. The air rate was regulated so that 2 kg of ore and 2 kg of charcoal were consumed every 10–12 minutes. For the first two charges, the ore to charcoal ratio was 1:2, and then increased to 1:1 (by weight) for the remainder of the smelt. The first tapping occurred after app. 2.5 hr, while the duration of the entire process, up to the removal of the bloom, was ca. 5 hr. (see summary in Table 2).

**Table 2:** Technical parameters and results of three experimental smelts.

	Smelting							Bloom Smithing		
Field Experiment	Total weight of roasted ore (Kg)	Total weight of charcoal (kg)	Ore/Charcoal ratio	Smelting duration	Bloom Weight (kg)	Yield bloom/ore (%)	Total slag weight (Kg)	Weight of quarter of bloom (kg)	Weight of forged bar (kg)*	Yield bar/bloom (%)
EXP-1 (IP-49)	32	48	0.7	04:45	5.7	16.9	17	0.88	0.41	46.6
EXP-5 (IP-43)	35	43	0.8	04:57	6.3	18.0	18	1.37	0.83	60.6
EXP-6 (IP-61)	38	50	0.8	05:10	7.3	19.2	21	1.89	1.20	63.5

* Bar was forged from ca. ¼ of the bloom (usually the smallest part).

** Yield was calculated in two stages, from ore to bloom and from bloom (= quarter) to bar.

### Osmium isotopic analysis

Samples of ores, roasted ores, and slag were prepared for osmium analysis by crushing about 10g of sample down to 0.5 mm to ensure best homogeneity. From these, 3g were further milled to grain size of <63 μm. Bloom and iron bar were prepared by filing, using a Dremel with a vidia tool to produce 1g.

Osmium isotope analyses were carried out following methods described previously in Brauns et al. [39,40]. Samples weighing 50–100 mg (from bloom and bar) and ca. 1g (all other sample materials), were weighed into pre-spiked (^190^Os tracer) Carius tubes, followed by dissolution and equilibration with inverse aqua regia at 240 °C. Osmium was extracted by distillation of the volatile tetroxide, condensed on a very small volume (20 μl) of chilled H_2_SO_4_ and then collected in 1.5 ml of 6.8 N HBr. Final purification of osmium was achieved by micro-distillation [41].

Os isotope ratios were measured by ion-counting on a modified Finnigan-MAT 261 mass spectrometer [42] operated in NTIMS mode [42] and corrected for mass bias and oxides [43]. Internal (2SD) precision for unknowns was <0.2%. Final ^187^Os/^188^Os ratios were corrected for blank (0.1–0.05 pg Os, (^187^Os/^188^Os) blank of 0.108), assuming an osmium yield of 85% [39]. Blank contribution for samples with low osmium concentrations are less than 0.5%. During the course of this study, JM-Os-DTM standard reference material yielded an average ^187^Os/^188^Os ratio of 0.17393 +/- 38 (n = 7), consistent with published results from other laboratories (e.g. two sets of long-term averages from DTM: 0.17429 & 55, 0.17396 & 38, [23]; two sets of averages from Monash University, Australia: 0.17367 & 58, 0.17400 & 21, [44,45]). In addition to this reference we conducted several full replica analyses in order to repeat the results using different sample amounts (see Table 3).

**Table 3:** Results of Os IC analysis. Os isotopes composition of experimental iron smelting; ores, blooms, bars, slag, and additional materials.

Experiment	Sample type	Sample name	^187^Os/^188^Os	2σ	Os ppt	2σ	Os ppt/Ore ppt
**EXP-1: IP49**	Ore IP49	IP-49	1.0437	0.0032	183	2	1.10
	Ore IP49	IP-21	1.0717	0.0032	179	9	1.07
	Ore IP49	IP-22	1.0591	0.0032	138	7	0.83
	**Average Ore**		1.0581	0.0032	167	6	1.00
	**Std Dev. Ore**						
	Roasted ore IP49	F.EXP-1-50	1.0497	0.0032	157	1	0.94
	Bloom	F.EXP-1-40	1.0460	0.0032	950	9	5.69
	Bar	F.EXP-1-43	1.0490	0.0032	1104	10	6.61
	Tap slag	F.EXP-1-16 (First tapping)	0.8415	0.0026	3	0	0.02
	Tap slag	F.EXP-1-22a (Last Tapping)	0.9265	0.0028	4	0	0.03
	Tap slag	F.EXP-1-22b (Last Tapping)	0.7701	0.0023	3	0	0.02
	Tap slag	F.EXP-1-22b replica	0.7947	0.0024	3	0	0.02
	Tap slag	F.EXP-1-22c (Last Tapping)	0.9467	0.0029	5	0	0.03
	Tap slag	F.EXP-1-22d (Last Tapping)	0.7351	0.0022	3	0	0.02
	Tap slag	F.EXP-1-22e (Last Tapping)	0.8945	0.0027	3	0	0.02
	**Average Tap Slag**		0.8442	0.0026	3	0	
	**Std Dev. Tap Slag**		0.0813				
	Furnace slag	F.EXP-1-24	1.0068	0.0031	200	2	1.20
	Smithing slag	F.EXP-1-45	1.0505	0.0032	155	1	0.93
**EXP-5: IP43**	Ore IP43	IP-43	1.1157	0.0034	5123	51	1.07
	Ore IP43	IP-43	1.0383	0.0031	4429	44	0.93
	**Average Ore**		1.0770	0.0033	4776	48	1.00
	**Std Dev. Ore**		0.0387	0.0001	347	3	
	Roasted ore IP43	F.EXP-5-50	1.0173	0.0031	6089	57	1.27
	Roasted ore IP43	F.EXP-5-50-replica	1.0145	0.0031	6667	62	1.40
	Bloom	F.EXP-5-40	1.0347	0.0031	69183	642	14.49
	Bloom	F.EXP-5-40 replica	1.0368	0.0031	48147	447	10.08
	Bar	F.EXP-5-43	1.0371	0.0031	133574	1240	27.97
	Bar	F.EXP-5-43 replica	1.0367	0.0031	129438	1201	27.10
	Tap slag	F.EXP-5-2 (first tapping)	1.0337	0.0031	213	2	0.04
	Tap slag	F.EXP-5-13 (last tapping)	1.0453	0.0032	64826	602	13.57
	Furnace slag	F.EXP-5-16	1.0284	0.0031	27173	252	5.69
	Furnace slag	F.EXP-5-16 replica	1.0305	0.0031	14452	134	3.03
	Smithing slag	F.EXP-5-45	1.0440	0.0032	1279	12	0.27
**EXP-6: IP61**	Ore IP61	F.EXP-6-1	1.0357	0.0031	18932	176	1.18
	Ore IP61	F.EXP-6-1 replica	1.0357	0.0031	14407	134	0.89
	Ore IP61	F.EXP-6-2	1.0255	0.0031	14983	150	0.93
	Ore IP61	IP-39	1.0221	0.0033	18791	188	
	**Average Ore**		1.0297	0.0032	16778	162	1.04
	**Std Dev. Ore**		0.0061	0.0001	2094	21	
	Roasted ore IP61	F.EXP-6-50	1.0296	0.0031	15067	140	0.94
	Roasted ore IP61	F.EXP-6-50 replica	1.0640	0.0032	13683	127	0.85
	Bloom	F.EXP-6-40	1.0500	0.0032	63680	591	3.95
	Bar	F.EXP-6-43	1.0490	0.0032	63995	640	3.97
	Bar	F.EXP-6-43 replica	1.0498	0.0032	63680	591	3.95
	Tap slag	F.EXP-6-5 (first tapping)	1.0371	0.0031	188	2	0.01
	Tap slag	F.EXP-6-19 (last tapping)	1.0375	0.0031	42621	411	2.65
	Furnace slag	F.EXP-6-21	1.0585	0.0032	50233	466	3.12
	Furnace slag	F.EXP-6-21 replica	1.0501	0.0032	12530	116	0.78
	Smithing slag	F.EXP-6-45	1.0445	0.0032			0.00
	**Average Negev Ore**		1.0550	0.0032	7240	72	
	**Std Dev. Negev Ore**		0.0238	0.0000	8587	80	
**MATERIALS**	Kaolin	F.EXP-1-52	0.5869	0.0018	19	0	
	Charcoal Type 1	F.EXP-1-51	0.3595	0.0036	3	0	
**AJLOUN ORE**	Ajloun ore	IP-27	2.0556	0.0062	142	1	
	Ajloun ore	IP-28	2.1378	0.0064	252	3	
	Ajloun ore	IP-64	1.6067	0.0049	134	1	
	Ajloun ore	IP-65	2.4945	0.0076	155	1	
	Ajloun ore	IP-66	2.1205	0.0064	277	3	
	**Average**		2.0830	0.0063	192	2	
	**Std Dev**.		0.2833	0.0009	60	1	

## Results

In order to substantiate the Os IC system for provenancing, previously suggested by Brauns et al. [15], a systematic approach was undertaken by initiating an experimental smelting of iron ores in which the technical part of the operational sequence (chaîne opératoire) of iron production could be followed and monitored, allowing the Os IC to be analyzed in a well-defined system.

**Fig 3:**
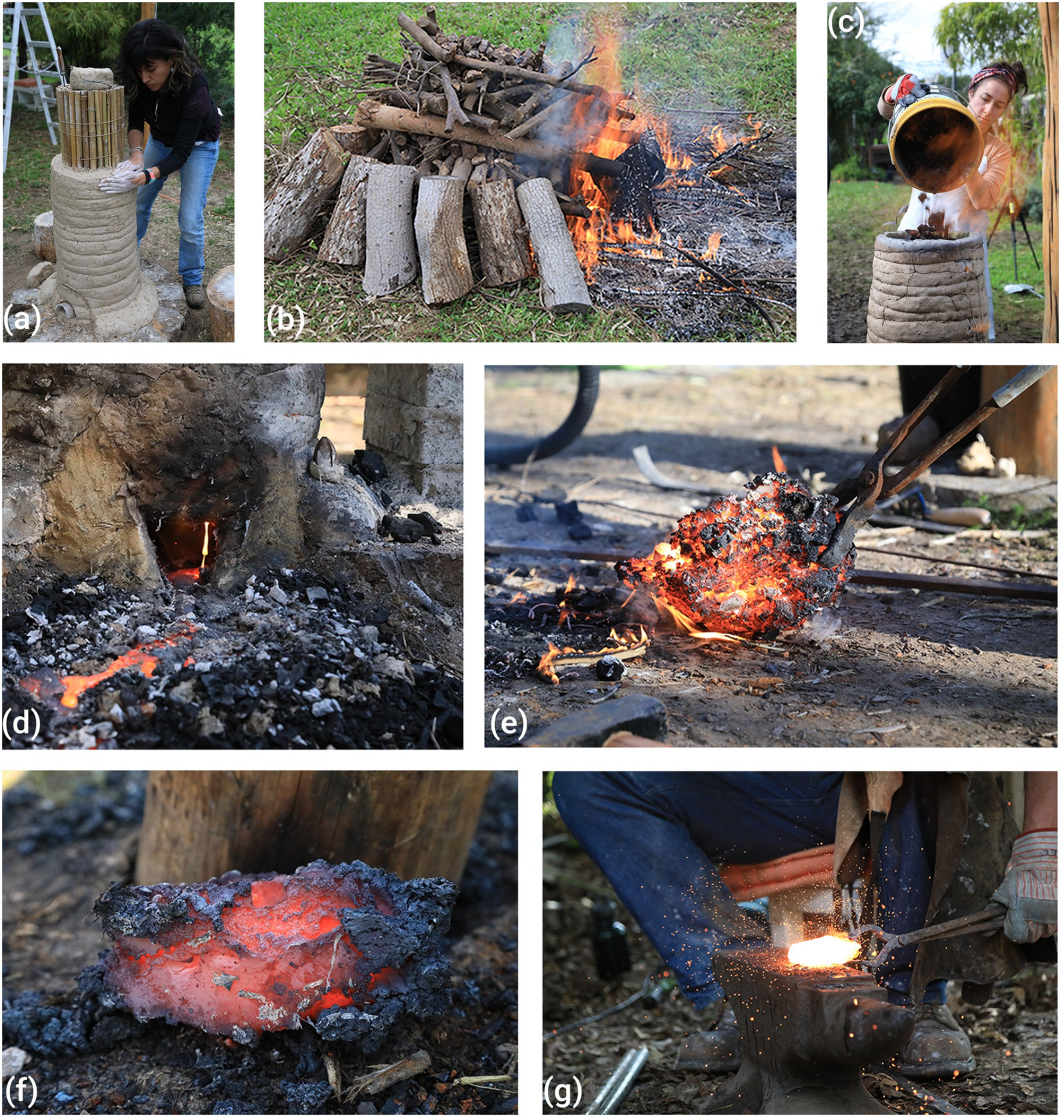
Various stages of field smelting experiments. (a) Building the shaft furnace using clay and bamboo, (b) Ore roasting, (c) Loading the furnace with charcoal during smelting, (d) Tapping the slag, (e) Removing the bloom from the furnace when red hot, (f) View of the bloom after being sectioned into four part while still red hot (g) Smithing the bar on an iron anvil.

**Fig 4:**
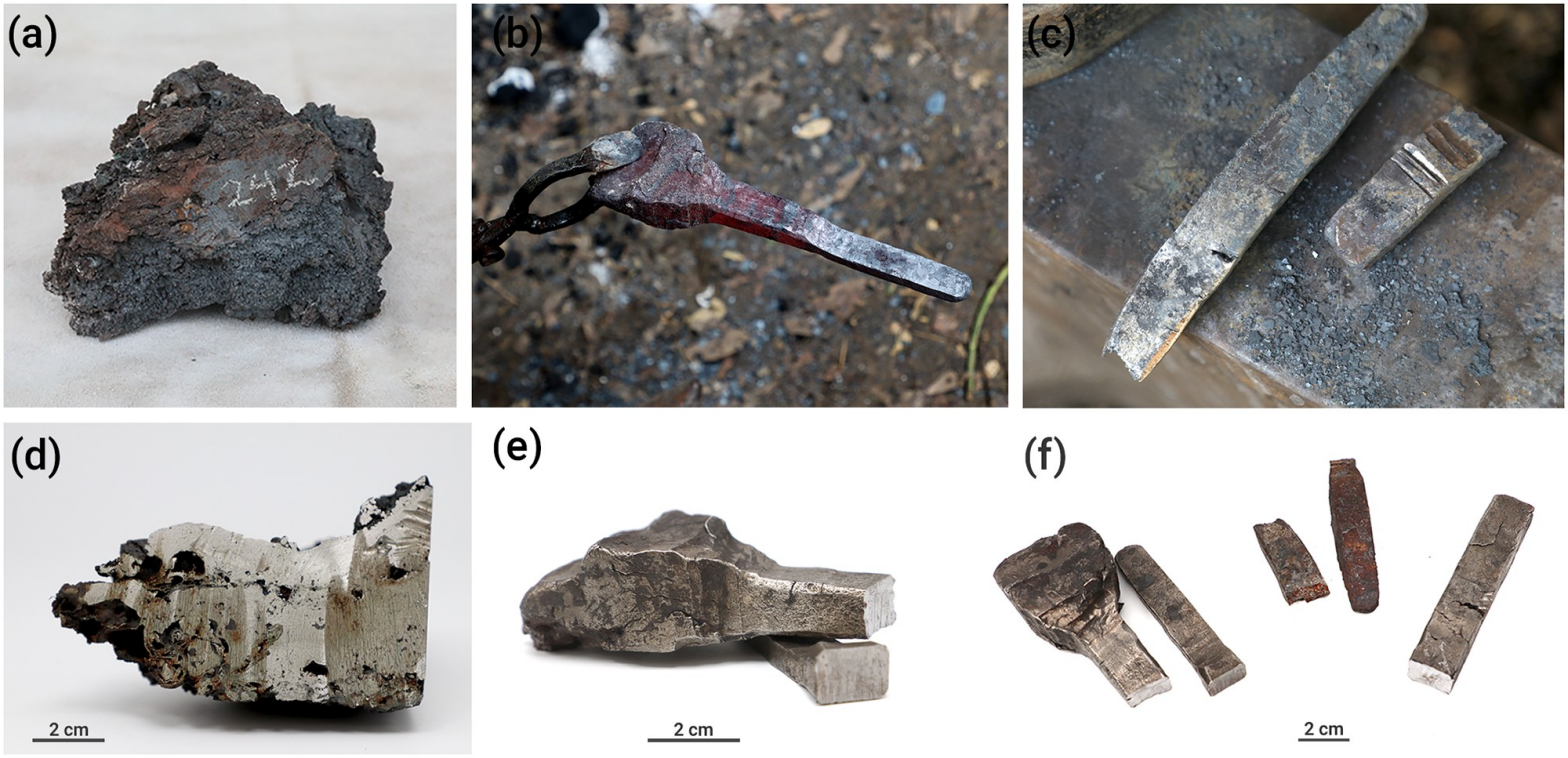
Bloom and bars from the experiments. (a) One quarter of the bloom prior to smithing (EXP-5, IP43) (b) Red hot bar (EXP-5, IP43), (c) Bar on anvil (EXP-6, IP61), (d) Quarter of the bloom after having been cut in the lab (EXP-5-IP43), (e and f) The bars after cutting for analysis.

Three successful smelting experiments using three different iron ore deposits, Zavar (IP49), Nekarot-Evus (IP43) and Nekarot (IP61) from locations in modern Israel were carried out in a bloomery shaft furnace (Fig 3). In each of these smelts, a bloom was produced, which was then forged into a bar (Figs 3 and 4). By conducting the full production process, we were able to show that the three ores chosen for the experiments were suitable for iron production in a bloomery furnace.

All the products and by-products formed in the smelting process were subjected to Os isotopic analysis. Table 2 provides a summary of the amount of raw materials involved in each experiment (minerals and charcoal), and the calculated yield. A cross section of the bloom and bar is seen in Fig 4.

**Fig 5:**
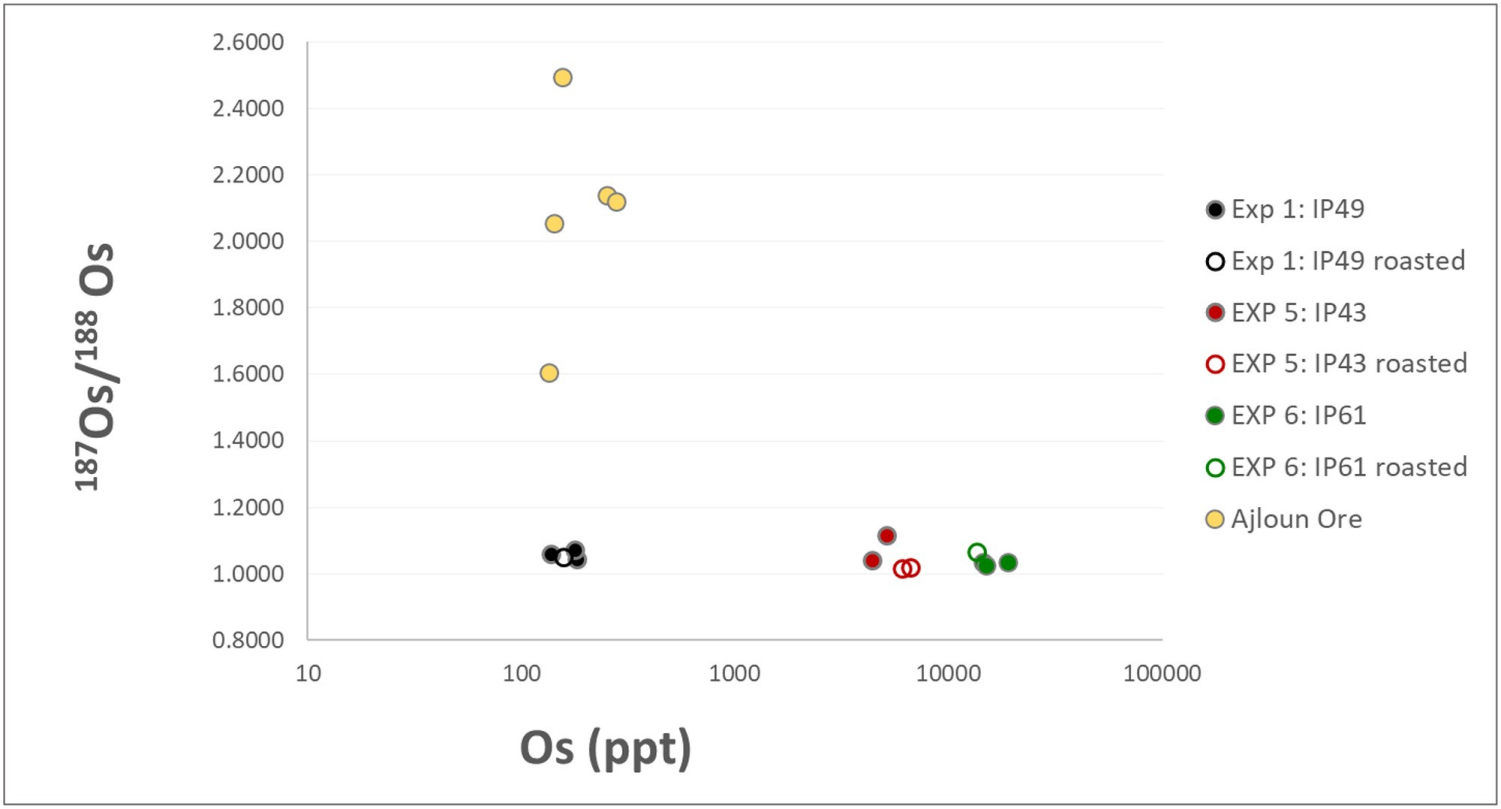
^187^Os/^188^Os ratios measured in iron ores. The ^187^Os/^188^Os ratio of iron ores used in the smelting experiments (before and after roasting), plotted against the osmium total concentration in ppt. Also shown for comparison are samples from the Ajloun ore deposits.

The osmium isotopic composition was measured for 48 samples (Table 3). These included the three ore sources—before and after beneficiation and roasting, three blooms and three bars, as well as by-products including tap slag, furnace slag and primary smithing slag. The ^187^Os/^188^Os ratio of the Negev ores which were used in the smelting experiments and the ore samples from *Mugharet el-Wardeh* in Ajloun, Jordan (analyzed for comparison in this study) are presented against their osmium concentration (in ppt) in Fig 5.

While the Negev ores share the same isotopic ratio and cannot be differentiated from one another based on the Os IC, the Ajloun ore reveals higher variability of the Os IC and a higher ^187^Os/^188^Os ratio, suggesting a more radiogenic ore source. Despite the fact that the three ore sources from the Negev share the same Os IC, there is a certain variation in the osmium concentrations which may be used as an additional discriminating factor to differentiate between the sources (and see more below).

**Fig 6:**
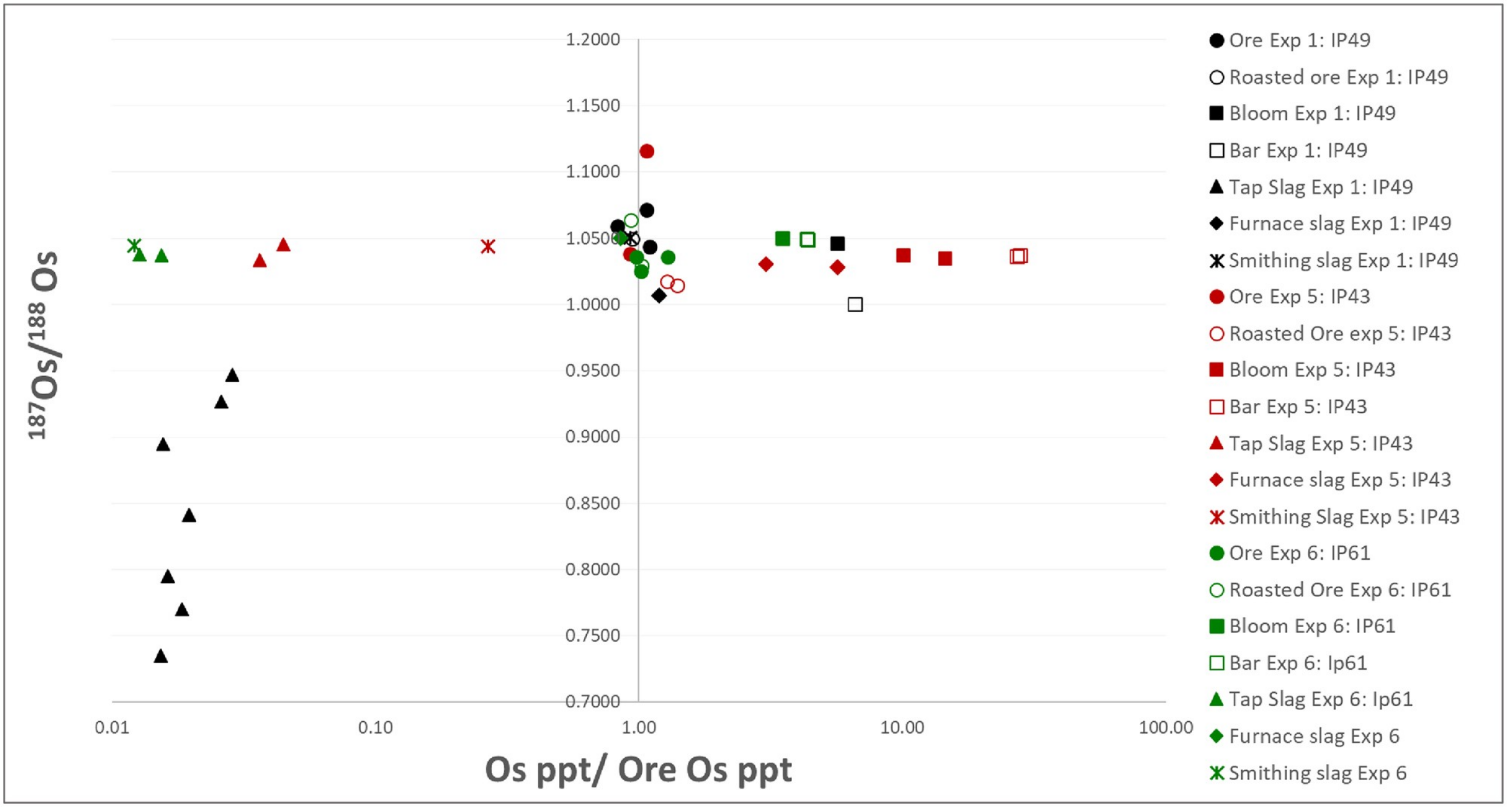
Osmium IC analysis of materials from the smelting experiments. Results of the ^187^Os/^188^Os ratio of iron ores, blooms, bars and slag produced in the three experiments, plotted against their osmium total concentration normalized to their ore osmium concentration, in ppt.

The ^187^Os/^188^Os ratio of the experimental blooms, bars and slag is plotted against the osmium concentration, normalized to the osmium concentration in the respective ore (Fig 6). This representation reveals strong enrichment of osmium into the reduced metal, i.e., the blooms and bars, and strong depletion of osmium in the silicate tap slag. Smithing slag shows variable osmium content, either similar or lower than those of the ores, while furnace slag is mostly enriched. The first and the last tap slags obtained from each experiment were measured. In addition, multiple analyses were repeated on the last tap slag from experiment 1 (F.EXP-1-22, Fig 6). The relatively high variability and the lower ^187^Os/^188^Os ratio obtained from the latter may be due to contamination from other materials used in the smelting process, such as charcoal and/or kaolin clay, which was used for building the shaft furnace. The charcoal has a very low osmium concentration of 3 ± 0.03 ppt with a ^187^Os/^188^Os ratio of 0.3595 ± 0.0036, which theoretically can lower the isotopic ratio in the slag. However, since tapped slag from the latter two experiments (EXP-5 and EXP-6) do not show a similar effect, we assume that the source of the contamination is the kaolin clay, which has a higher osmium concentration of 19 ± 0.18 ppt and ^187^Os/^188^Os ratio of 0.5869 ± 0.0018 (Table 3). Naturally the clay, releasing its osmium content mostly during the first firing of the furnace, would have more of an effect on the slag formed in the first experiment. Admittedly, the lower concentration of osmium in the ore smelted in the first experiment may have caused the slag to be more prone to contamination from the furnace material.

Several main conclusions may be drawn:

1. The ^187^Os/^188^Os ratio remains constant throughout the *chaîne opératoire*–from ore to bloom to the final product, with no significant fractionation. This observation, together with clear differences in Os IC of ores from different regions (southern Israel and northwestern Jordan) forms the basis for the possibility of using osmium to provenance iron in the southern Levant and beyond.
2. Varying concentrations of osmium in different ores may be used as an additional criterion to differentiate between ore sources.
3. The enrichment of osmium into the bloom and metal in comparison to the parent ore and, on the other hand, the depletion of osmium in some types of slag, raises the possibility that in some cases osmium concentrations may be used to differentiate between different by-products, and hence different stages of the bloomery process.
4. The strong depletion of osmium in silicate slag (and especially tap slag) suggests that this type of slag is more prone to contamination and therefore less suited for determining the iron ore source based on this method.

## Discussion

The analysis of Os IC from three consecutive iron smelting experiments showed no significant fractionation between the ore, the bloom and the bar. This confirms Brauns et al.’s [15] hypothesis according to which the Os IC will not be affected by the high temperature reduction process. Another pertinent conclusion was the significant variability of ^187^Os/^188^Os ratios between ore deposits in different geological settings; in our case, a major difference was identified between the *Mugharet el-Wardeh*, *Ajloun* ore deposit (2.083 ± 0.283) and that of the Negev ores (1.0552 ± 0.0233). Analysis of a larger dataset of iron ores sources in the region is currently underway.

When overlapping ^187^Os/^188^Os ratios exist between various ore deposits it may be possible to use the osmium concentration as another discriminating tool. From the data presented here, it is clear that even though some ore deposits, formed at roughly similar geological ages, may have the same isotopic ratio–they can considerably differ in their osmium concentrations (the three Negev deposits show a 3 order of magnitude difference in their osmium ppt levels).

Considering that osmium is a highly siderophile element, it is expected to partition into the metal phase in a metal-silicate system, which is the case in the process of iron smelting. Indeed, we can detect strong depletion of osmium in the slag phase (especially tap slag, see below) in comparison to the enrichment of osmium in the metal, bloom and bar. Consequently, when analyzing a final product (an iron artifact), theoretically, its osmium concentration could not be significantly lower than the iron ore used. This principle can be used to rule out certain deposits as possible ore sources and has enormous potential for sourcing ancient iron finds.

From the data presented here, we conclude that Os IC is particularly suitable for the provenancing of metallic artifacts, be it a bloom, bar or final object, and to a lesser extent to the provenancing of slag. Tap slag is generally the most prevalent by-product of the bloomery process and can be easily identified visually in the archaeological record. However, as it is characteristically rich in silica and therefore does not typically contain unreacted ore, its osmium concentrations are particularly low. This renders this type of slag much more prone to contamination from associated materials used in the process, as was shown in the results of experiment 1 (FEXP-1, IP49). Osmium concentration is as low as 3 ppt, which is less than two percent of its concentration in the ore. This means that any minute contamination from other materials (in this case kaolinite clay and/or charcoal) is likely to significantly affect the ^187^Os/^188^Os ratio. On the other hand, furnace slag, more difficult to identify and less abundant in the archaeological record, does incorporate some unreacted/non-reduced ore and is therefore better suited for provenance. Preliminary analysis showed that it is also composed of a significant amount of reduced metal that did not agglomerate into bloom, similar to what is sometimes termed ‘crown material’ and thus was enriched by osmium [46]. It is therefore recommended that any slag considered for Os IC provenance will be thoroughly investigated metallographically to determine its mineralogy, composition and formation processes (the slag and metal from the above experiments are currently under study). Moreover, to rule out possible contamination, it is necessary that any associated materials, such as furnace structures and fragments, will be also sampled for comparative analysis.

Notably, while the aim of this study to prove the feasibility of Os IC analysis for the provenancing of ancient iron was reached, future work is necessary in order to compile a database of Os IC of relevant iron ore sources, in any specific region of interest. In addition, the sampling of a large number of iron objects is the next step for examining the applicability of the method in various parts of the ancient world. Despite preliminary results that show that corrosion does not alter the Os IC of ancient artefacts (unpublished), the subject requires further investigation.

The authors of this paper are currently conducting a survey of iron ores in the southern Levant. Concomitantly, iron artifacts from well stratified and controlled contexts from Iron Age sites are being subjected for Os IC analyses as well.

## Summary and conclusions

The results of the systematic analyses presented here show great promise for Os IC as a viable tool for the provenacing of ancient iron. The results show that the ^187^Os/^188^Os ratio is preserved from ore to metal, with no isotopic fractionation. Significantly, enrichment/depletion of Os content were observed between ore to metal and ore to slag. This has major implications for our ability to distinguish between different slag types and also suggests which of the production remains are better suited for this type of analysis. Finally, the natural variability in ^187^Os/^188^Os ratio and osmium concentration found among different ore sources in the southern Levant indicated that Os IC analysis is a powerful tool for the provenance of archaeological ferrous metals, at least in this region. Future work will include a systematic analysis of additional ores and iron objects from various Iron Age sites in the region.
