# Correction: Osmium isotope analysis as an innovative tool for provenancing ancient iron: A systematic approach

**DOI:** 10.1371/journal.pone.0316588

**Published:** 2024-12-26

**Authors:** Michael Brauns, Naama Yahalom-Mack, Ivan Stepanov, Lee Sauder, Jake Keen, Adi Eliyahu-Behar

In Table 3, there are errors in the rows Std Dev. Ore and Std Dev. Tap Slag of EXP-1: IP49, Tap slag (last tapping) of EXP-5: IP43, Ore IP61 (IP-39) and Tap slag (last tapping) of EXP-6: IP61 and Ajloun Ore. Please see the correct Table 3 here.

**Table 3:** Results of Os IC analysis. Os isotopes composition of experimental iron smelting; ores, blooms, bars, slag, and additional materials.

Experiment	sample type	Sample name	187Os/188Os	2s	ppt Os	2s	Os ppt/Ore ppt
**EXP-1: IP49**	Ore IP49	IP-49	1.0437	0.0032	183	2	1.10
	Ore IP49	IP-21	1.0717	0.0032	179	9	1.07
	Ore IP49	IP-22	1.0591	0.0032	138	7	0.83
	**Average Ore**		1.0581	0.0032	167	6	1.00
	**Std Dev. Ore**		0.0140	0	20	3	
	Roasted ore IP49	F.EXP-1-50	1.0497	0.0032	157	1	0.94
	Bloom	F.EXP-1-40	1.0460	0.0032	950	9	5.69
	Bar	F.EXP-1-43	1.0490	0.0032	1104	10	6.61
	Tap slag	F.EXP-1-16 (First tapping)	0.8415	0.0026	3	0	0.02
	Tap slag	F.EXP-1-22a (Last Tapping)	0.9265	0.0028	4	0	0.03
	Tap slag	F.EXP-1-22b (Last Tapping)	0.7701	0.0023	3	0	0.02
	Tap slag	F.EXP-1-22b replica	0.7947	0.0024	3	0	0.02
	Tap slag	F.EXP-1-22c (Last Tapping)	0.9467	0.0029	5	0	0.03
	Tap slag	F.EXP-1-22d (Last Tapping)	0.7351	0.0022	3	0	0.02
	Tap slag	F.EXP-1-22e (Last Tapping)	0.8945	0.0027	3	0	0.02
	**Average Tap Slag**		0.8442	0.0026	3	0	0.02
	**Std Dev. Tap Slag**		0.0813	0.0002	0.9	0	
	Furnace slag	F.EXP-1-24	1.0068	0.0031	200	2	1.20
	Smithing slag	F.EXP-1-45	1.0505	0.0032	155	1	0.93
**EXP-5: IP43**	Ore IP43	IP-43	1.1157	0.0034	5123	51	1.07
	Ore IP43	IP-43	1.0383	0.0031	4429	44	0.93
	**Average Ore**		1.0770	0.0033	4776	48	1.00
	**Std Dev. Ore**		0.0387	0.0001	347	3	
	Roasted ore IP43	F.EXP‐5‐50	1.0173	0.0031	6089	57	1.27
	Roasted ore IP43	F.EXP‐5‐50-replica	1.0145	0.0031	6667	62	1.40
	Bloom	F.EXP‐5‐40	1.0347	0.0031	69183	642	14.49
	Bloom	F.EXP‐5‐40 replica	1.0368	0.0031	48147	447	10.08
	Bar	F.EXP‐5‐43	1.0371	0.0031	133574	1240	27.97
	Bar	F.EXP‐5‐43 replica	1.0367	0.0031	129438	1201	27.10
	Tap slag	F.EXP‐5‐2 (first tapping)	1.0337	0.0031	174	2	0.04
	Tap slag	F.EXP‐5‐13 (last tapping)	1.0453	0.0032	213	2	0.04
	**Average Tap slag**		1.0395	0.0032	194	2	0.04
	**Std Dev. Tap Slag**		0.0058	0.0000	20	0	
	Furnace slag	F.EXP‐5‐16	1.0284	0.0031	27173	252	5.69
	Furnace slag	F.EXP‐5‐16 replica	1.0305	0.0031	14452	134	3.03
	Smithing slag	F.EXP‐5‐45	1.0440	0.0032	1279	12	0.27
**EXP-6: IP61**	Ore IP61	F.EXP‐6‐1	1.0357	0.0031	18932	176	1.18
	Ore IP61	F.EXP‐6‐1 replica	1.0357	0.0031	14407	134	0.89
	Ore IP61	F.EXP-6-2	1.0255	0.0031	14983	150	0.93
	Ore IP61	IP-39	1.0221	0.0033	18791	188	1.12
	**Average Ore**		1.0297	0.0032	16778	162	1.04
	**Std Dev. Ore**		0.0061	0.0001	2094	21	
	Roasted ore IP61	F.EXP-6-50	1.0296	0.0031	15067	140	0.94
	Roasted ore IP61	F.EXP-6-50 replica	1.0640	0.0032	13683	127	0.85
	Bloom	F.EXP-6-40	1.0500	0.0032	63680	591	3.95
	Bar	F.EXP-6-43	1.0490	0.0032	63995	640	3.97
	Bar	F.EXP-6-43 replica	1.0498	0.0032	63680	591	3.95
	Tap slag	F.EXP-6-5 (first tapping)	1.0371	0.0031	227	2	0.01
	Tap slag	F.EXP-6-19 (last tapping)	1.0375	0.0031	188	2	0.01
	**Average slag**		1.0373	0.0031	208	2	0.01
	**Std Dev. Tap Slag**		0.0002	0.0000	20	0	
	Furnace slag	F.EXP-6-21	1.0585	0.0032	50233	466	3.12
	Furnace slag	F.EXP-6-21 replica	1.0501	0.0032	12530	116	0.78
	Smithing slag	F.EXP-6-45	1.0445	0.0032			0.00
	**Average Negev Ore**		1.0550	0.0032	7240	72	
	**Std Dev. Negev Ore**		0.0238	0.0000	8587	80	
Materials	Kaolin	F.EXP-1-52	0.5869	0.0018	19	0	
	Charcoal Type 1	F.EXP-1-51	0.3595	0.0036	3	0	
Ajloun Ore	Ajloun ore	IP-27	2.0556	0.0062	142	1	0.74
	Ajloun ore	IP-28	2.1378	0.0064	252	3	1.31
	Ajloun ore	IP-63	1.6067	0.0049	134	1	0.70
	Ajloun ore	IP-64	2.4945	0.0076	155	1	0.81
	Ajloun ore	IP-65	2.1205	0.0064	277	3	1.44
	**Average**		2.0830	0.0063	192	2	1.00
	**Std Dev.**		0.2833	0.0009	60	1	
